# Efficient polymalic acid production from corn straw hydrolysate by detoxification of phenolic inhibitors

**DOI:** 10.3389/fbioe.2023.1339982

**Published:** 2023-12-13

**Authors:** Jun Xia, Zhongyang Qiu, Shibiao Ma, Qianqian Liu, Renxian Han, Xiaoyan Liu, Jiaxing Xu

**Affiliations:** Jiangsu Key Laboratory for Biomass-Based Energy and Enzyme Technology, Jiangsu Collaborative Innovation Center of Regional Modern Agriculture and Environmental Protection, College of Chemistry and Chemical Engineering, Huaiyin Normal University, Huaian, China

**Keywords:** poly(malic acid), phenolic inhibitors, microbial conversion, detoxification, resin H103

## Abstract

Inhibitory compounds generated from lignocellulose pretreatment would inhibit Poly (malic acid) (PMA) production by *Aureobasidium pullulans*, but the tolerance mechanism of *A. pullulans* to lignocellulosic inhibitor is poorly understood. In this study, the cellular response of *A. pullulans* to lignocellulosic inhibitor stress was studied. Among the three groups of inhibitors (furans, weak acids and phenolic aldehydes), phenolic aldehyde was the dominant inhibitor for PMA production. Phenolic aldehyde was mainly converted into phenolic alcohol by *A. pullulans*, and phenolic alcohol also exhibited severe inhibition on PMA production. Furthermore, the effect of detoxification methods on inhibitor-removal and PMA fermentation was investigated, both CaCO_3_ and overliming presented poor detoxification effect, whereas resin H103 could remove both furan derivatives and phenolic compounds efficiently, thereby producing 26.27 g/L of PMA with a yield of 0.30 g/g in batch fermentation. This study will be beneficial for the development of PMA production from lignocellulosic biomass.

## Introduction

Inhibitory compounds generated during pretreatment of lignocellulosic biomass is a serious challenge for the biorefinery process (Bellido et al., 2011; Franden et al., 2013). The lignocellulosic inhibitors include furan derivatives (furfural and 5-hydromethylfurfural [HMF]), weak organic acids (acetic acid and formic acid) and phenolic compounds (4-hydroxybenzaldehyde, vanillin, syringaldehyde, etc.) (Ujor and Okonkwo, 2022; Ebrahimian et al., 2023). Furfural and HMF are generated by the degradation of pentose and hexose, respectively, acetic acid is resulted from the hydrolysis of acetylxylan in hemicellulose, and phenolic compounds are formed due to the hydrolysis of lignin during the pretreatment process (Liu et al., 2021). The inhibitory compounds not only decrease cellulase activity in the saccharification step, but also inhibit cell metabolism in the fermentation step (Ruchi et al., 2021; Jiang et al., 2023). Therefore, elucidating and alleviating the negative effects of inhibitors is necessary for the efficient conversion of lignocellulose to biochemicals.

*Aureobasidium pullulans* is a biotechnologically important fungus due to its ability of producing various valuable chemicals, including polymalic acid (PMA), pullulan, siderophore and extracellular enzymes (Chi et al., 2009). Among these bioproducts, PMA is a versatile biopolymer with great potential application in pharmaceuticals and foods (Zou et al., 2019). For example, PMA is biocompatible, water-soluble and non-immunogenic, thus this biopolymer is a promising nanovector for drug delivery system (Loyer and Cammas-Marion, 2014). Meanwhile, PMA can be easily hydrolyzed into malic acid, which is an acidulant in food industry (Chi et al., 2016). To decrease the cost of raw materials, the exploration of lignocellulosic waste for PMA production has been strongly stimulated, efficient PMA production from different lignocellulosic biomass (wheat straw, corncob, barley straw, bagasse, etc.) has been successfully conducted (Zou et al., 2015; Cao et al., 2019; Cao et al., 2020a; Yegin et al., 2019), and the cellular response of *A. pullulans* to lignocellulosic inhibitor stress is a hot research topic. For example, Zou et al. (2015) reported that the cell growth of *A. pullulans* CCTCC M2012223 was completely inhibited by the individual addition of HMF (3 g/L), furfural (0.5 g/L), acetic acid (2 g/L) and formic acid (0.5 g/L). However, Yegin et al. (2019) found that the cell growth of *A. pullulans* NRRL Y-2311–1 was not affected by the individual addition of HMF (2 g/L), furfural (1 g/L) and acetic acid (7.5 g/L), and PMA production was just slightly decreased under these stresses. Similarly, Cao et al. (2019, 2020a) also reported that the presence of HMF (0.5 g/L) and acetic acid (2.5 g/L) did not show negative effects on PMA production and cell growth. In general, the researchers have concentrated on the influence of furans and weak acids on PMA production, but the effect of phenolic compounds on PMA fermentation are rarely studied. This phenomenon is possibly due to: (i) The concentration of phenolic compounds are much lower than furan derivatives and weak acids, thus phenolic compounds were not considered to be the major inhibitors. (ii) Various phenolic compounds existed in the lignocellulosic hydrolysate, including phenolic aldehydes, alcohols and acids. The variety of phenolic compounds makes it difficult to elucidate the mechanism of phenolic compounds on PMA production. However, phenolic compounds usually exhibit stronger inhibition on fermenting strains than furans and weak acids, even though the concentration of phenolic inhibitors is low (Mills et al., 2009). Therefore, it is necessary to explore the effect of phenolic compounds on PMA production, and the results may provide insights into the studies about PMA fermentation from lignocellulose.

The presence of inhibitory compounds in the lignocellulosic hydrolysate would severely disturb cell metabolism, thereby inhibiting the utilization of sugars and preventing product formation. To overcome this problem, two strategies have been proposed: (i) Development of robust strains with enhanced tolerance against toxic compounds via evolutionary adaptation. For example, the parental strain of *A. pullulans* CCTCC M2012223 could not grow under the stress of HMF, furfural or acetic acid, thus the aerobic fibrous bed bioreactor was employed for evolutionary adaptation and an adapted strain was isolated, which could overcome the inhibition and improve PMA production (Zou et al., 2015), similar strategy was also conducted to improve pullulan production by *A. pullulans* CCTCC M2012259 (Wang et al., 2014). (ii) Application of detoxification treatments to lignocellulosic hydrolysate. Yegin et al. (2019) reported that the egg shell, which is mainly composed of CaCO_3_, could act as a detoxifying agent for PMA production. Cao et al. (2020a) found that PMA production was significantly enhanced using membrane technology as a detoxification method. Unfortunately, the relationship between inhibitor-removal and PMA production was not clear in these studies, it is difficult to elucidate the relationship between inhibitor-removal and PMA production. On the other hand, macroporous adsorption resins are non-ionic resins capable of recovering non-polar or less polar organic compounds from aqueous solutions. Recent studies indicated that macroporous adsorption resins are able to remove furan derivatives and phenolic compounds based on adsorption with van der Waals interactions (Ijzer et al., 2015; Li et al., 2020). It is promising to detoxify the lignocellulosic hydrolysate by macroporous resins for PMA production.

In this study, the aim was to identify the dominant inhibitor for PMA production and to minimize its negative effects. Meanwhile, the removal efficiency of lignocellulosic inhibitors with different detoxification methods was expounded. Moreover, efficient PMA production from resin-detoxified corn straw hydrolysate was successfully conducted.

## Materials and methods

### Reagents and enzymes

The macroporous resin H103 was purchased from the Chemical Factory of Nankai University (Tianjin, China), the specific surface area and the average pore diameter was 900–1,100 m^2^/g and 8.5–9.0 nm (data from the manufacturer), the resin was soaked with ethanol for 24 h, 2% (v/v) HCl and 2% (w/v) NaOH for 2 h, respectively. Then the resin was washed to neutral pH and dried at 40°C in vacuum. The commercial cellulase CTec2 was purchased from Novozyme (Beijing, China), the filter paper activity was 201.2 FPU/mL and the total protein concentration was 82.4 mg/mL. All other chemicals were of analytical grade and purchased from Macklin Biochemical Technology Co., Ltd. (Shanghai China).

### Raw material and biorefinery processing

Corn straw was harvested from Lianyungang, Jiangsu, China, in autumn 2022. The corn straw contained 34.93% ± 0.29% cellulose and 18.94% ± 1.90% hemicellulose, and the dry matter content of the corn straw was 91.23% ± 0.34%. The corn straw was coarsely chopped, washed, dried and milled to pass through a 10-mm mesh. The corn straw was pretreated with diluted sulfuric acid solution (2%, v/v) at a solid to liquid ratio of 1:9 (w/v), and the pretreatment was carried out by autoclaving at 121°C for 60 min. After cooling down to room temperature, the mixture was filtered to obtain the liquid and solid fractions, respectively. The liquid fraction was detoxified by different methods, and the solid fraction was washed to neutral pH and dried to a constant weight. Then the solids were added into the detoxified acid hydrolysate, and the cellulase CTec2 was added into the mixture at an enzyme loading of 20 mg protein/g cellulose. The enzymatic hydrolysis was carried out at 50°C for 72 h, and the liquid fraction was separated by vacuum filtration. Furthermore, the liquid fraction was concentrated in vacuum at 70°C to achieve a total sugar concentration of approximately 100 g/L.

The acid hydrolysate was detoxified by CaCO_3_, overliming and resin H103, respectively. The procedure was as follows: (i) CaCO_3_ and resin H103: the pH value of the acid hydrolysate was adjusted to 5.0 with the addition of sodium hydroxide, then CaCO_3_ and resin H103 was respectively added, the dosage of CaCO_3_ and H103 resin was 3% and 5% (w/v), respectively. Subsequently, the mixture was shaken at 150 rpm for 2 h at 30°C, the liquid fraction was obtained by centrifugation. (ii) Overlimimg: the acid hydrolysate was adjusted to pH 10.0 with the addition of Ca(OH)_2_, and the mixture was heated at 60°C for 2 h. Subsequently, the pH value was adjusted to 5.0 using sulfuric acid, and the liquid fraction was obtained by centrifugation.

### Microorganism and culture methods

The PMA-producing strain *A. pullulans* HA-4D (CGMCC No. 7.208) was maintained on potato dextrose agar slants. The seed medium was composed of 80 g/L glucose, 1 g/L yeast extract, 1 g/L NaNO_3_, 0.1 g/L KH_2_PO_4_, 0.1 g/L MgSO_4_, 0.5 g/L KCl and 20 g/L CaCO_3_. For seed culture, a loop of *A. pullulans* HA-4D was inoculated into the seed medium and aerobically cultured at 25°C for 48 h. The synthetic medium for PMA fermentation contained (g/L): glucose 100, NaNO_3_ 2, KH_2_PO_4_ 0.1, MgSO_4_ 0.1, ZnSO_4_ 0.1, KCl 0.5 and CaCO_3_ 30. When corn straw hydrolysate was employed for PMA production, the corn straw hydrolysate was used instead of glucose, and the rest of the components are the same as the synthetic medium. CaCO_3_ and corn straw hydrolysate were autoclaved separately and mixed together after cooling to room temperature. Shake-flask fermentation was conducted in 250 mL shake flasks containing 50 mL fermentation medium, 10% (v/v) of *A. pullulans* seed culture was inoculated into the fermentation medium, the culture was carried out at 25°C and 200 rpm.

### Analytical methods

The concentration of glucose, xylose, HMF, furfural and acetic acid in the hydrolysate was determined by an HPLC apparatus (Agilent 1,260, United States) equipped with an Aminex HPX-87H column (Bio-rad, United States) and a refractive index detector, the HPLC analysis was carried out using 5 mM H_2_SO_4_ as the eluent with a flow rate of 0.6 mL/min at 65°C. The phenolic inhibitors were analyzed by an HPLC method using an Alphasil VC-C18 column (5 μm, 4.6 × 250 mm, Acchrom-Tech, China), 0.1% formic acid (phase A) and acetonitrile (phase B) served as the mobile phases, and the gradient elution was carried out according to a previous study (Luo et al., 2021), the HPLC analysis was performed at 0.8 mL/min and 35°C, the phenolic aldehydes and acids were detected at 280 nm, and phenolic alcohols were monitored at 223 nm. The total soluble phenol in the hydrolysate was determined using Folin-Ciocalteu reagent, and the analysis was carried out as previously described (Yu et al., 2020). The analysis of PMA and dry cell weight (DCW) was performed according to our previous study (Xia et al., 2022).

## Results and discussion

### Effect of lignocellulosic inhibitors on cell growth and PMA production

**FIGURE 1:**
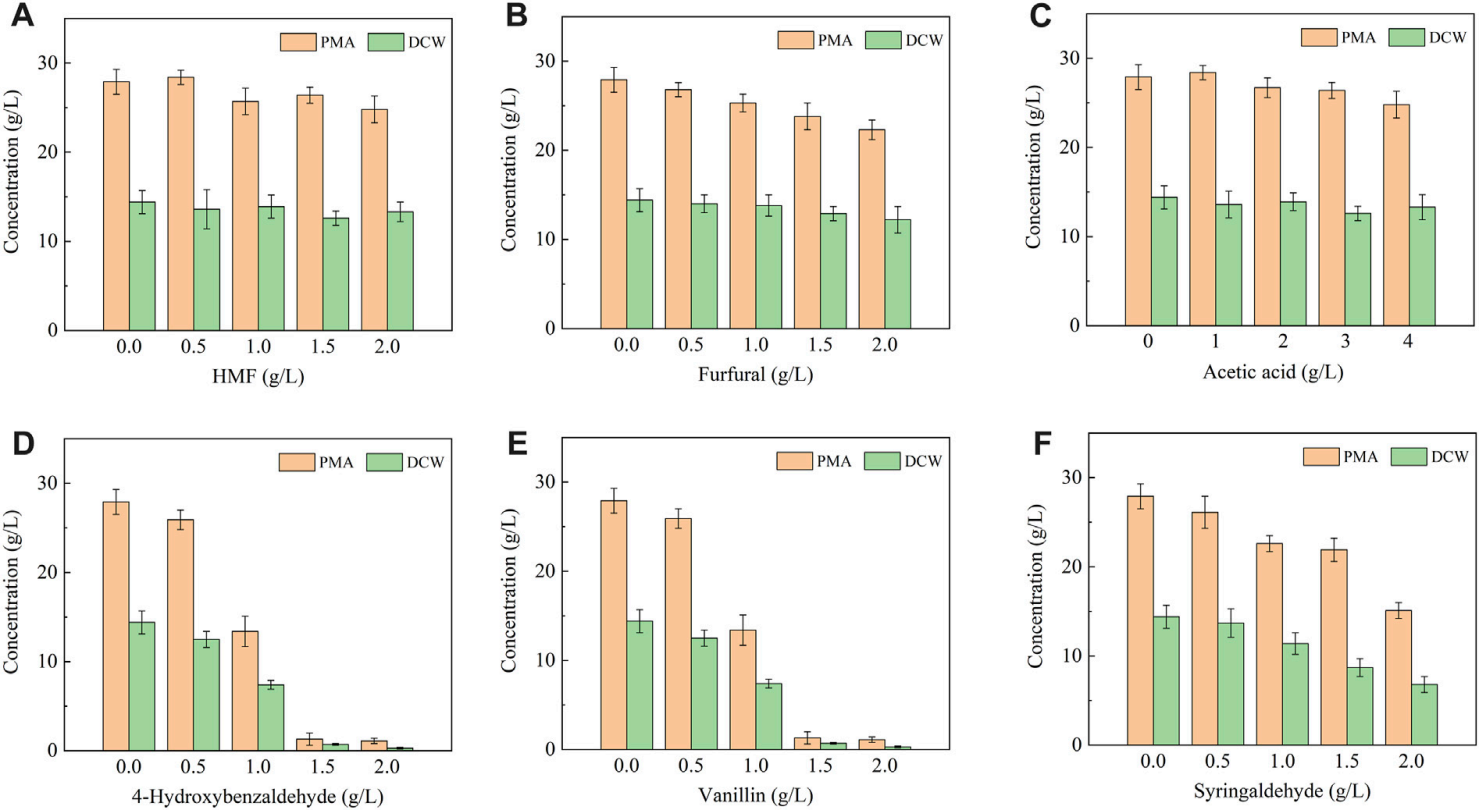
Effect of lignocellulosic inhibitors on cell growth and PMA production by *A. pullulans* HA-4D. The inhibitors included HMF **(A)**, furfural **(B)** acetic acid **(C)**, 4-hydroxybenzaldehyde **(D)**, vanillin **(E)** and syringaldehyde **(F)**. The inhibitors were individually added into the medium, and shake-flask fermentation was carried out at 25°C, 200 rpm for 8 days.

The inhibitors generated during the pretreatment process can be divided into three groups: furan derivatives (furfural and HMF), weak acids (acetic acid) and phenolic compounds. 4-Hydroxybenzaldehyde, vanillin and syringaldehyde are three typical phenolic aldehydes of lignocellulosic hydrolysate, which are considered as lignin derivatives of hydroxyphenyl group (H), guaiacyl group (G) and syringyl group (S), respectively (Xu et al., 2019; Cai et al., 2021). Therefore, the above six compounds were selected as typical lignocellulosic inhibitors and used in this study. As shown in Figure 1, *A. pullulans* HA-4D displayed strong tolerance to acetic acid, HMF and furfural under the tested dosage. Acetic acid did not show obvious inhibition effect on cell growth and PMA production within 0–4 g/L, which was probably attributed to the neutralization effect of CaCO_3_ in the fermentation medium. Meanwhile, PMA production was not significantly affected by HMF or furfural, the concentration of PMA was decreased by 5.4% and 14.6% with the addition 1.5 g/L of HMF and furfural, respectively. These results were in good agreement with previous studies (Cao et al., 2019; 2020a; Yegin et al., 2019). By contrast, *A. pullulans* HA-4D exhibited weak tolerance to phenolic aldehydes under the tested dosage, thereby giving a poor cell growth and PMA production. The concentration of PMA was decreased by 96.1%, 96.8% and 45.9% in the presence of 2 g/L of 4-hydroxybenzaldehyde, vanillin and syringaldehyde, respectively. Therefore, furans and weak acids did not inhibit cell growth and PMA biosynthesis, but phenolic aldehydes resulted in significant decrease on biomass formation and PMA production.

**FIGURE 2:**
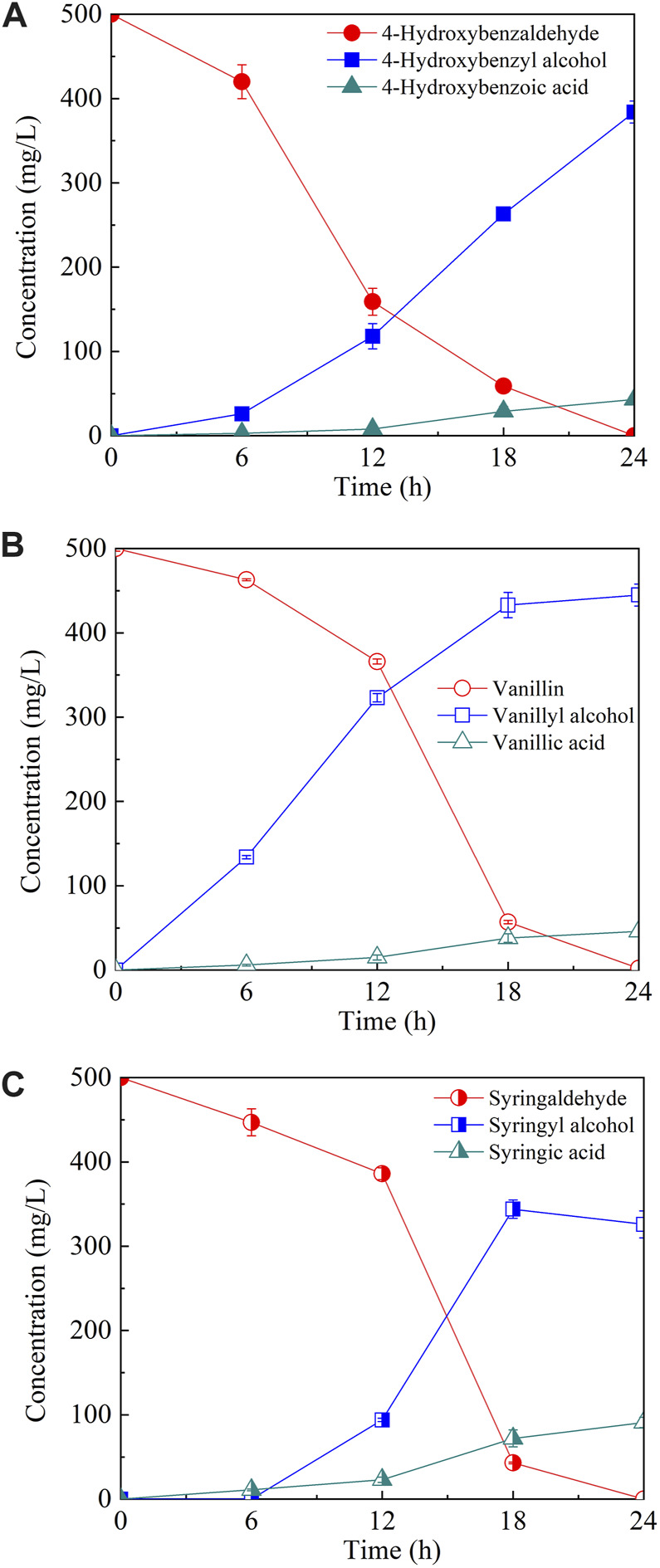
The microbial conversion of phenolic aldehydes by *A. pullulans* HA-4D. The phenolic aldehydes included 4-hydroxybenzaldehyde **(A)**, vanillin **(B)** and syringaldehyde **(C)**. 0.5 g/L of phenolic aldehydes were individually added into the medium at the beginning of the fermentation. Shake-flask fermentation was carried out at 25°C, 200 rpm for 24 h.

**FIGURE 3:**
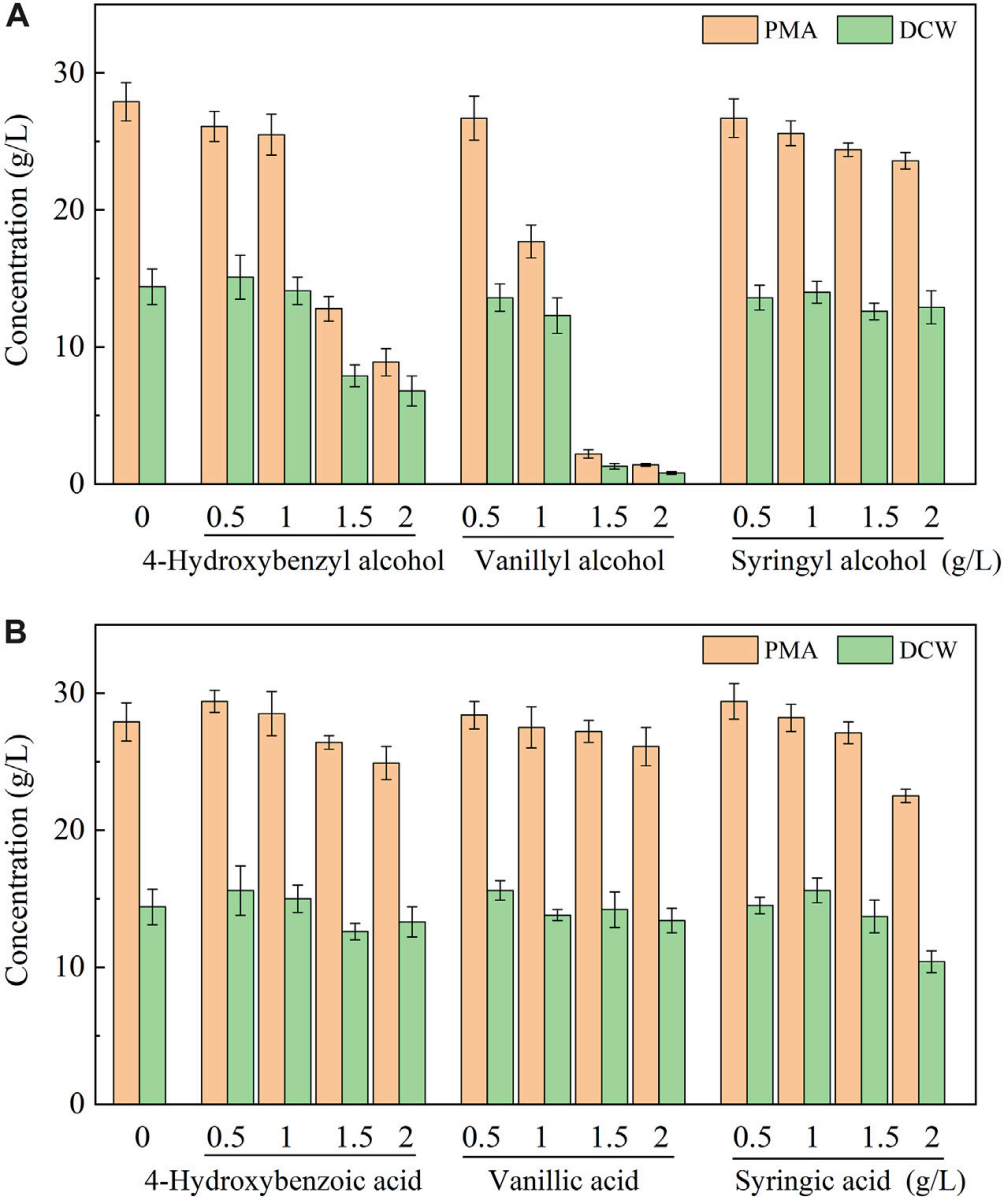
Effect of phenolic alcohols **(A)** and phenolic acids **(B)** on cell growth and PMA production by *A. pullulans* HA-4D. 0.5–2 g/L of phenolic alcohols and acids were individually added into the medium at the beginning of the fermentation. Shake-flask fermentation was carried out at 25°C, 200 rpm for 8 days.

To elucidate the tolerance mechanism of *A. pullulans* HA-4D, 500 mg/L of phenolic aldehyde inhibitors were individually added into the medium, and the bioconversion products were analyzed. As shown in Figure 2, all the phenolic aldehydes were completely converted into the corresponding alcohols and acids within 24 h, 384 mg/L of 4-hydroxybenzyl alcohol, 445 mg/L of vanillyl alcohol and 344 mg/L of syringyl alcohol were respectively accumulated. Meanwhile, 43 mg/L of 4-hydroxybenzoic acid, 46 mg/L of vanillic acid and 91 mg/L of syringic acid were also obtained. It turned out that phenolic alcohols were the main products of microbial conversion. To verify if phenolic alcohol or acid derivatives were inhibitory for PMA production, the above six compounds were respectively added into the medium. As shown in Figure 3, both cell growth and PMA production was not significantly affected by phenolic acids within the dosage of 0–2 g/L, indicating that phenolic acids were not toxic to *A. pullulans* HA-4D. By contrast, 4-hydroxybenzyl alcohol and vanillyl alcohol exhibited strong inhibition on PMA production, the concentration of PMA was decreased by 68.1% and 95.0% in the presence of 2 g/L of 4-hydroxybenzyl alcohol and vanillyl alcohol, respectively. However, the alcohol derivatives of syringaldehyde presented slight inhibition on PMA biosynthesis within 2 g/L. In summary, PMA production was severely inhibited by phenolic aldehydes, which were mainly converted into alcohol derivatives by *A. pullulans* HA-4D, and 4-hydroxybenzyl alcohol and vanillyl alcohol also exhibited strong inhibition on PMA biosynthesis, this may explain why *A. pullulans* HA-4D behaved poor PMA production when phenolic compounds were present in the lignocellulosic hydrolysate.

Furthermore, considering that various inhibitors were simultaneously present in the practical lignocellulosic hydrolysate, the synergistic effect of inhibitors should be considered. PMA fermentation medium contained 30 g/L of CaCO_3_, which could neutralize acetic acid toxicity, thus acetic acid was not included in the combination of inhibitors, and the synergistic effect of furans and phenolic aldehydes on PMA production was investigated. As shown in Table 1, 0.5 g/L of HMF and furfural were simultaneously added into the medium in run I, while 4-hydroxybenzaldehyde, vanillin and syringaldehyde were additionally supplemented in run II ∼ IV. In run I, the concentration of PMA, DCW and residual glucose were comparable to those of control, indicating that the combination of HMF and furfural did not inhibit PMA production and cell growth. However, the amount of PMA was significantly decreased when phenolic aldehydes were added into furans, PMA concentration was decreased to 10.58 g/L, 2.02 g/L and 0.97 g/L when 4-hydroxybenzaldehyde, vanillin and syringaldehyde were added, respectively. Meanwhile, both cell growth and glucose consumption were severely inhibited under these stresses. Given that the inhibitory effect was related to the dosage of inhibitors, 0.75 g/L of each of HMF and furfural were used in run V, it turned out that PMA production, cell growth and sugar consumption were much better than those of run I ∼ IV. Consequently, the presence of furans alone did not inhibit PMA production, but the simultaneous existence of furans and phenolic aldehydes caused severe inhibition on PMA biosynthesis, thus phenolic aldehydes were the dominant inhibitors for PMA production, and the removal of phenolic aldehydes should be the first priority on selecting detoxification methods.

**TABLE 1:** Synergistic effect of lignocellulosic inhibitors on PMA production and cell growth by *A.pullulans* HA-4D.

	PMA (g/L)	DCW (g/L)	Residual glucose (g/L)	Productivity (g/L/h)
Control	26.08 ± 1.57	13.76 ± 0.40	5.58 ± 0.16	0.136
Run I	25.59 ± 0.53	11.23 ± 0.63	5.86 ± 0.75	0.133
Run II	10.58 ± 1.06	3.44 ± 0.62	59.05 ± 5.06	0.055
Run III	2.02 ± 0.28	1.23 ± 0.11	89.18 ± 2.42	0.011
Run IV	0.97 ± 0.12	0.54 ± 0.11	96.20 ± 2.59	0.005
Run V	17.75 ± 0.72	9.92 ± 0.21	13.74 ± 1.36	0.092

The shake-flask fermentation was conducted at 25℃, 200 rpm for 8 days, the synthetic medium containing 100 g/L of glucose was utilized for the fermentation. The control group did not contain any inhibitor.

Run I: 0.5 g/L furfural + 0.5 g/L HMF.

Run II: 0.5 g/L furfural + 0.5 g/L HMF + 0.5 g/L 4-hydroxybenzaldehyde.

Run III: 0.5 g/L furfural + 0.5 g/L HMF + 0.5 g/L vanillin.

Run IV: 0.5 g/L furfural + 0.5 g/L HMF + 0.5 g/L syringaldehyde.

Run V: 0.75 g/L furfural + 0.75 g/L HMF.

### Effect of detoxification method on the removal of inhibitor

Corn is the most produced grain in the world, the estimated global yield of corn is approximately 1.1 billion tons in 2022 (https://www.statista.com). China is the second largest country for producing corn, about 300 million tons of crop straws are generated in China per year, which is generally discarded or burned in the field (Wang et al., 2020a), thus corn straw was selected as the typical lignocellulosic substrate for PMA production in this study. Considering that diluted acid pretreatment can hydrolyze hemicellulose into xylose in the liquid fraction while keeping cellulose in the solid fraction, thus this method was applied to prepare corn straw hydrolysate in this study. The effect of acid dosage on the properties of liquid and solid fraction of corn straw hydrolysate was tested (Supplementary Table S1). When 2% (v/v) H_2_SO_4_ was used, the amount of glucose released from the solid fraction was the highest, and the sugar content (2.44 g/L of glucose plus 18.75 of g/L xylose) in the liquid fraction was also relatively high, thus 2% (v/v) H_2_SO_4_ was chosen in the subsequent experiments.

Meanwhile, the acid hydrolysate derived from 2% (v/v) H_2_SO_4_ also contained various inhibitory compounds. 3.59 g/L of acetic acid was detected in the acid hydrolysate, which was the most abundant inhibitor. 0.12 g/L of HMF and 0.36 g/L of furfural were also accumulated. The concentration of 4-hydroxybenzaldehyde, vanillin and syringaldehyde was 12.29 mg/L, 3.49 mg/L and 10.71 mg/L, respectively. To verify if detoxification should be applied to corn straw hydrolysate prior to PMA fermentation, the acid hydrolysate was used for PMA production without any detoxification treatment. It turned out that only 2.55 g/L of PMA was produced from 22.92 g/L of total sugar, thus the detoxification of lignocellulosic hydrolysate is urgently needed to improve PMA production.

Next, the effect of detoxification method on the removal of inhibitor was investigated. Overliming and macroporous resin were employed since they are commonly used in previous studies. In addition, PMA fermentation medium contained 30 g/L CaCO_3_, Yegin et al. (2019) reported that egg shell (94%–97% CaCO_3_) could act as a detoxifying agent for PMA production, but the detailed information about inhibitor-removal was not reported, thus the detoxification effect of CaCO_3_ was also tested. As shown in Table 2, CaCO_3_ displayed a poor effect on the removal of toxic compounds, the concentration of all the tested inhibitors was just slightly decreased. Overliming was effective on the removal of furans, approximately 50% of HMF and furfural was removed, whereas the amount of the rest of inhibitors was comparable to that of CaCO_3_. On the other hand, resin H103 exhibited an excellent detoxification effect, HMF, furfural, vanillin and syringaldehyde were completely removed, and the concentration of 4-hydroxybenzaldehyde was decreased from 12.29 mg/L to 5.34 mg/L. Considering that the contents of 4-hydroxybenzaldehyde, vanillin and syringaldehyde in the acid hydrolysate were low, the total soluble phenol (TSP) was employed to assay the removal efficiency of phenolic compounds (Yu et al., 2020). 410.49 mg/L of TSP was detected in the control group, and the concentration of TSP was decreased by 5.4%, 11.4% and 67.4% when CaCO_3_, overliming and resin H103 was utilized, respectively. Among the three detoxification methods, resin H103 gave the best performance on the removal of toxic inhibitors, both furans and phenolic compounds were efficiently removed. Meanwhile, sugar loss also occurred after detoxification treatment, and the extent of sugar loss was positively related to the removal of inhibitors, the total sugar was decreased by 1.9%, 6.0% and 9.6% when CaCO_3_, overliming and resin H103 was utilized, respectively.

**TABLE 2:** Effect of detoxification method on PMA production and removal of inhibitors.

Detoxification	HMF	Furfrual	Acetic acid	4-Hydroxyben-	Vanillin	Syringaldehyde	TSP ^#^	Total Sugar	PMA production
method	(g/L)	(g/L)	(g/L)	zaldehyde (mg/L)	(mg/L)	(mg/L)	(mg/L)	(g/L)	(g/L)
Control ^*^	0.12 ± 0.002	0.36 ± 0.01	3.59 ± 0.08	12.29 ± 0.13	3.49 ± 0.32	10.71 ± 0.47	410.49 ± 4.10	22.92 ± 0.37	2.55 ± 0.19
CaCO_3_	0.12± 0.002	0.33 ± 0.02	3.48 ± 0.02	11.83 ± 0.25	3.31 ± 0.13	8.25 ± 0.52	388.88 ± 2.61	22.48 ± 0.09	2.82 ± 0.09
Overliming	0.06 ± 0.003	0.19 ± 0.01	3.41 ± 0.04	11.41 ± 0.19	2.89 ± 0.15	8.94 ± 0.17	363.55 ± 4.84	21.55 ± 0.41	3.31 ± 0.09
Resin H103	NA ^**^	NA	3.10 ± 0.21	5.34 ± 0.04	NA	NA	133.72 ± 5.96	20.71 ± 0.46	5.91 ± 0.35

* The acid hydrolysate of corn straw served as control, which was adjusted to pH 5.0 with NaOH and no other detoxification treatment was applied, CaCO_3_ was also not added into the fermentation medium; ** NA, not available; # TSP, total soluble phenol.

CaCO_3_ is an important component of PMA fermentation medium, and CaCO_3_ plays an important role as a stimulatory agent for PMA biosynthesis, which is mainly featured in the following aspects: (i) PMA synthetase is a non-ribosomal peptide synthetase (NRPS), the expression of PMA synthetase is activated in the presence of Ca^2+^ via a transcriptional activator Crz1 in the Ca^2+^ signaling pathway (Wang et al., 2020b). (ii) The transcriptome analysis revealed that the genes involved in energy production and conversion in *A. pullulans* strains were downregulated when CaCO_3_ was added into the medium, which may cause the redistribution of metabolic flux and more metabolic flux was directed to biosynthesis of PMA (Cao et al., 2020b). From the viewpoint of detoxification, the action mechanism of CaCO_3_ and overliming should be the same, the toxic inhibitors were adsorbed to the gypsum which was formed by the addition of CaCO_3_ or Ca(OH)_2_, the difference lies in that the reaction with CaCO_3_ was carried out at room temperature, while overliming was heated at 60°C, and the removal efficiency of inhibitors was much higher when overliming was conducted at higher temperature (Martinez et al., 2000). Yegin et al. (2019) reported that the detoxification and fermentation were carried out simultaneously when CaCO_3_ was utilized, thus the detoxification experiments using CaCO_3_ was conducted at room temperature in this study. Unfortunately, the present study indicated that neither CaCO_3_ nor overliming could remove phenolic compounds effectively, which was probably due to the low water solubility and hydrophobicity of phenolic compounds (Gu et al., 2015). By contrast, resin H103 exhibited a strong ability of removing furans and phenolic compounds, and the resulting sugar loss was also within an acceptable range, thereby giving the highest PMA production.

### PMA production from resin-detoxified corn straw hydrolysate

**FIGURE 4:**
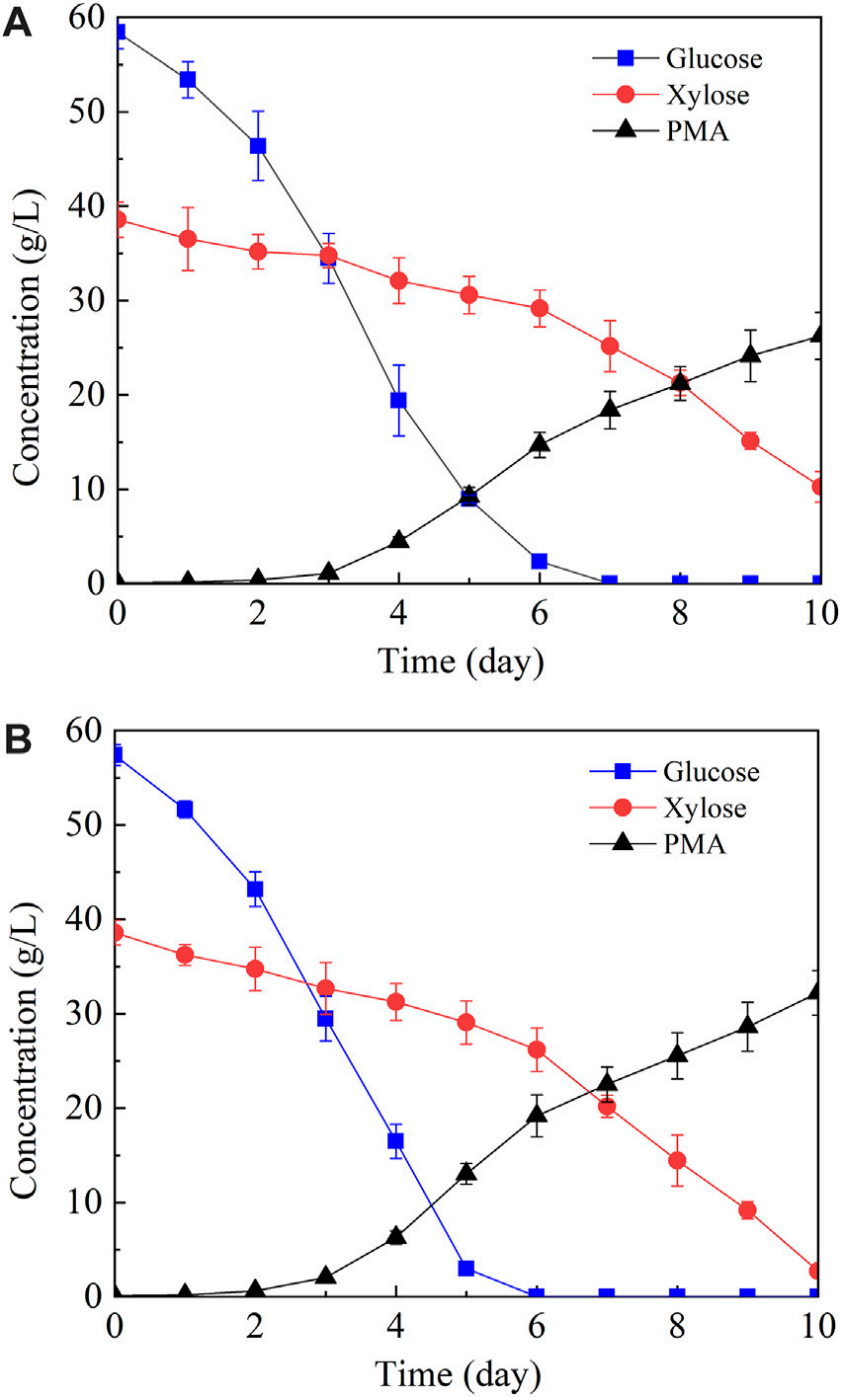
PMA production from corn straw hydrolysate **(A)** and artificial hydrolysate **(B)**. Both corn straw hydrolysate and artificial hydrolysate contained 58.4 g/L of glucose and 38.6 g/L of xylose. Shake-flask fermentation was carried out at 25°C, 200 rpm for 10 days.

In the above study, 5.91 g/L of PMA was produced from the resin-detoxified acid hydrolysate which contained 20.71 g/L of total sugar. It is well known that over-supply of carbon source is a prerequisite for PMA production by *A. pulllulnas* (Zou et al., 2019), thus the sugar content of corn straw hydrolysate should be increased to obtain a higher PMA yield. For this purpose, the resin-detoxified acid hydrolysate was mixed with the solid residues of pretreated corn straw, and the mixture was subjected to cellulase hydrolysis. 29.2 g/L of glucose and 19.3 g/L of xylose was obtained, the hydrolysate was then concentrated in vacuum and the concentration of glucose and xylose was increased to 58.4 g/L and 38.6 g/L, respectively. An artificial hydrolysate to mimic the sugar composition of corn straw hydrolysate was also utilized for PMA production in parallel. As shown in Figure 4, *A. pullulans* HA-4D consumed sugars sequentially, glucose was firstly utilized and xylose was consumed secondly. When corn straw hydrolysate was used, glucose was exhausted and the concentration of residual xylose was 10.65 g/L, 26.27 g/L of PMA with a yield of 0.30 g/g was obtained. By contrast, the concentration of PMA and residual xylose was 32.22 g/L and 2.73 g/L when the artificial hydrolysate was utilized. More PMA and higher sugar consumption rate was obtained with the artificial hydrolysate, suggesting that PMA production was slightly affected by the remaining toxic inhibitors in the corn straw hydrolysate.

**FIGURE 5:**
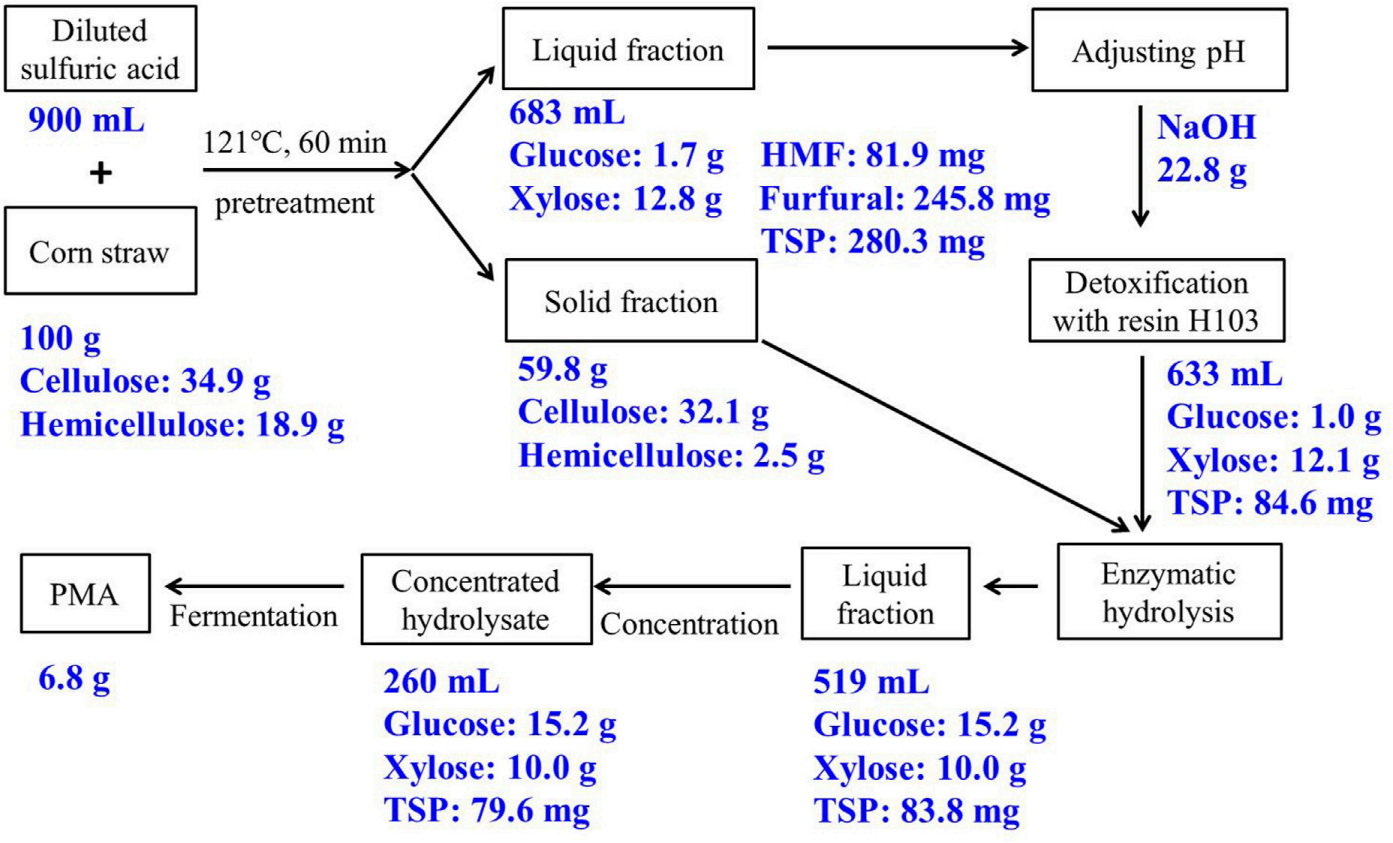
Mass balance in main steps for PMA production from corn straw. Abbreviations: HMF, 5-hydromethylfurfural; TSP, total soluble phenol.

The overall mass balance for PMA production from corn straw was shown in Figure 5. The sugar loss during biorefinery of corn straw lied in three steps: (i) After the diluted acid pretreatment of 100 g of corn straw, 37.3 g of glucose equivalent and 15.6 g of xylose equivalent were obtained, 12.1% of total sugar was lost in water-washing or formation of HMF/furfural. (ii) After resin-detoxification, 1.0 g of glucose and 12.1 g of xylose were recovered in the acid hydrolysate, 2.3% of total sugar was lost by resin-adsorption. (iii) After enzymatic hydrolysis, 15.2 g glucose and 10.0 g xylose were recovered, 43.8% of total sugar was lost in the solid fraction of corn straw. The majority of sugar was lost in the enzymatic hydrolysis, which was due to the insufficient pretreatment caused by the low heating temperature (121°C). On the other hand, furfural and HMF were completely removed by resin detoxification, and 70% of TSP was removed from corn straw hydrolysate, and the remaining TSP was slightly decreased after vacuum concentration. Overall, 25.2 g of sugar was obtained from 100 g of corn straw, and 6.8 g of PMA was finally produced, the corn straw hydrolysate by resin-detoxification is an adequate carbon source for PMA production.

Currently, it is controversial whether detoxification treatment should be applied to lignocellulosic hydrolysate prior to PMA fermentation. Zou et al. (2015, 2016) reported that the corncob hydrolysate could be directly used for PMA production without detoxification treatment, whereas other researchers reported that active carbon, egg shell or membrane technology should be applied to lignocellulosic hydrolysate (Yegin et al., 2019; Cao et al., 2020a). The different strains used in those studies are responsible for this contradiction. The present study indicated that PMA production by *A. pullulans* HA-4D was severely hindered by phenolic compounds, thus detoxification is an indispensable step and resin H103 exhibited an excellent detoxification effect. Resin H103 is an important polymeric resin with polystyrene skeleton, this resin has been successfully utilized for the treatment of phenolic wastewater and aniline-containing wastewater (Shan et al., 2015). Resin H103 has no functional groups, the effective removal of inhibitors is due to its large surface areas (900–1,100 m^2^/g) and van der Waals forces, which forms weak bonds with inhibitory compounds (Ijzer et al., 2015). The macroporous or mesoporous pores in the resins would provide channels to adsorb inhibitory molecules and mainly contributed to the strong adsorption capacity. For example, recovery of vanillin from aqueous solution was successfully conducted with resin H103, a maximum adsorption capacity of 416 mg/g (vanillin/resin) was achieved (Zhang et al., 2008). In this study, resin H103 provided the best performance of removing inhibitors, thereby giving efficient PMA production from corn straw hydrolysate, thus resin H103 is an effective adsorbent for the detoxification of lignocellulosic hydrolysate.

## Conclusion

Among the three groups of lignocellulosic inhibitors (furans, weak acids and phenolic aldehydes), phenolic aldehydes were the dominant inhibitors for PMA production. Phenolic aldehydes were mainly converted into phenolic alcohols by *A. pullulans* HA-4D, and phenolic alcohols also exhibited severe inhibition on PMA production. Therefore, the removal of phenolic compounds is the first priority on selecting detoxification methods. The detoxification effect of CaCO_3_, overliming and macroporous resin was tested, resin H103 could remove both furans and phenolic compounds efficiently, and 26.27 g/L of PMA with a yield of 0.30 g/g was obtained in batch fermentation. This study will be beneficial for the development of PMA production from lignocellulosic biomass.

## Data Availability

The original contributions presented in the study are included in the article/Supplementary Material, further inquiries can be directed to the corresponding author.
