# Supplementary material for: Efficient polymalic acid production from corn straw hydrolysate by detoxification of phenolic inhibitors
Source: Front Bioeng Biotechnol. 2023 Dec 13;11:1339982. doi: 10.3389/fbioe.2023.1339982 (PMC10751350; doi:10.3389/fbioe.2023.1339982)
Supplement: Supplementary file 1 [file Table1.docx]

**Supplementary materials for**

Efficient polymalic acid production from corn straw hydrolysate by detoxification of phenolic inhibitors

Jun Xia, Zhongyang Qiu, Shibiao Ma, Qianqian Liu, Renxian Han, Xiaoyan Liu, Jiaxing Xu^*^

*Jiangsu Key Laboratory for Biomass-Based Energy and Enzyme Technology,* *Jiangsu Collaborative Innovation Center of Regional Modern Agriculture and Environmental Protection, College of Chemistry and Chemical Engineering, Huaiyin Normal University, Huai’an 223300, China.*

**Correspondence author: Jiaxing Xu, xujiaxing@hytc.edu.cn*

**Table S1** The composition of liquid (a) and solid fraction (b) of corn straw after diluted sulfuric acid pretreatment.

| 1. Liquid fraction * | | | | | | | | |
| --- | --- | --- | --- | --- | --- | --- | --- | --- |
| Acid dosage (%, v/v) | Glucose  (g/L) | Xylose  (g/L) | HMF  (g/L) | Furfrual  (g/L) | Acetic acid  (g/L) | 4-Hydroxybenzaldehyde (mg/L) | Vanillin  (mg/L) | Syringaldehyde  (mg/L) |
| 3.0 | 2.62 ± 0.26 | 16.15 ± 2.20 | 0.15 ± 0.04 | 0.32 ± 0.06 | 3.00 ± 0.24 | 17.14 ± 1.10 | 13.33 ± 0.69 | 11.61 ± 0.33 |
| 2.0 | 2.44 ± 0.16 | 18.75 ± 1.26 | 0.11 ± 0.03 | 0.36 ± 0.01 | 3.59 ± 0.08 | 12.29 ± 0.13 | 3.49 ± 0.32 | 10.71 ± 0.47 |
| 1.0 | 2.41 ± 0.35 | 19.29 ± 2.90 | 0.12 ± 0.01 | 0.27 ± 0.01 | 3.20 ± 0.07 | 10.65 ± 0.13 | 6.14 ± 0.45 | 8.55 ± 0.99 |
| 0.5 | 1.33 ± 0.10 | 16.63 ± 0.77 | 0.06 ± 0.01 | 0.09 ± 0.01 | 2.43 ± 0.02 | 9.82 ± 0.06 | 10.66 ± 0.22 | 9.50 ± 0.99 |
| 0.2 | 0.27 ± 0.05 | 0.94 ± 0.14 | 0.03 ± 0.01 | 0.02 ± 0.004 | 0.36 ± 0.03 | 8.74 ± 0.31 | 0.79 ± 0.06 | 2.80 ± 0.17 |

* The corn straw was pretreated with 2% (v/v) H_2_SO_4_ solution at a solid to liquid ratio of 1:9 (w/v) at 121 ℃ for 60 min. After cooling down to room temperature, the mixture was filtered to obtain the liquid and solid fractions, respectively.

| 1. Solid fraction |  |  |  |  |
| --- | --- | --- | --- | --- |
| Acid dosage  (%, v/v) | Solid recovery  (%) | Cellulose  (%) | Hemicellulose  (%) | Saccharification Yield (%)* |
| 3.0 | 62.18 ± 0.45 | 53.17 ± 2.05 | 5.93 ± 0.10 | 55.55 ± 2.21 |
| 2.0 | 59.86 ± 0.36 | 53.60 ± 1.47 | 4.20 ± 0.15 | 63.71± 1.99 |
| 1.0 | 63.65 ± 0.98 | 51.29 ± 3.47 | 3.88 ± 0.25 | 60.39 ± 5.57 |
| 0.5 | 67.92 ± 1.07 | 49.53 ± 2.04 | 8.99 ± 1.28 | 59.77 ± 0.78 |
| 0.2 | 81.44 ± 0.30 | 41.75 ± 1.70 | 18.52 ± 2.13 | 40.72 ± 4.58 |
| Raw corn straw | / | 34.93 ± 0.29 | 18.94 ± 1.90 | / |

*The saccharification was carried out in the mixture of 10 mL citrate buffer (50 mM, pH 4.8), 0.5 g pretreated corn straw and cellulase CTec2 (20 mg protein/g cellulose), the mixture was incubated at 50℃, 150 rpm for 72 h. The saccharification yield was calculated by the ratio of the amount of glucose released in the hydrolysate to the theoretical glucose derived from cellulose content in pretreated corn straw.
